# 

**Published:** 1907-08

**Authors:** 


					﻿PUBLISHED MONTHLY.	ATLANTA	SUBSCRIPTION $1.00
JOURNAL-RECORD S
SURGEON GENERAL’S OFFICET U , ■
of Medicine 1
(Atlanta Medical and Surgical Journal, Established 1855. / rn i 1/
and Southern Medical Record, Established 1870.	\ JuU iCul
Vol. IX.	Atlanta, Ga., August, 1907.	No. 5?
				

